# A Soft Capacitive Pressure Sensor Based on a Liquid Dielectric Layer

**DOI:** 10.3390/s25092700

**Published:** 2025-04-24

**Authors:** Meng Zhang, Chengjie Qiu, Jianxiang Wang, Xuan Huang, Wu Zhang, Lip-Ket Chin, Wenli Shang

**Affiliations:** 1 School of Electronics and Communication Engineering, Guangzhou University, Guangzhou 510006, China; zhangmeng@gzhu.edu.cn (M.Z.);; 2 Key Laboratory of On-Chip Communication and Sensor Chip, Guangdong Higher Education Institutes, Guangzhou 510006, China; 3 School of Physics and Material Science, Guangzhou University, Guangzhou 510006, Chinazhangwu@gzhu.edu.cn (W.Z.); 4 Department of Electrical and Electronic Engineering, The Hong Kong Polytechnic University, Hong Kong SAR 999077, China

**Keywords:** capacitive pressure sensor, soft electronics, adjustable sensitivity

## Abstract

Soft electronic technology has broad application prospects in biomedical and wearable devices, among others, due to its flexibility, lightweight nature, and biocompatibility. Although various materials and structures have been proposed for pressure sensors based on soft electronic technology, most studies focus on a specific function with fixed sensitivity, lacking tunability to expand the operational range. In this work, we demonstrated a low-cost polydimethylsiloxane (PDMS)-based pressure sensor that can be easily fabricated by laser ablation and mature PDMS fabrication technology. We then employed a liquid solution to serve as the dielectric layer of the pressure sensor. By injecting different liquid solutions, the sensitivity of the capacitive pressure sensor can be easily adjusted. A 2.73-fold increase in sensitivity and excellent sensing linearity with a determination coefficient greater than 0.85 were achieved. The pressure sensor was applied to demonstrate material property measurements and Morse code adaptation. We foresee that the adjustable soft capacitive pressure sensor has extensive applications in wearable devices, material metrology, healthcare point-of-care devices, and other fields.

## 1. Introduction

Due to its unique flexibility and broad application prospects, soft electronic technology has become a research hotspot nowadays [1,2,3,4,5,6,7]. Unlike traditional electronics, which are built on rigid substrates such as silicon or printed circuit boards, soft electronics are constructed on stretchable, rollable, or malleable substrates. Consequently, soft electronics offer advantages such as being lightweight, conformable, bio-compatible, etc., making them suitable for applications in wearable devices, the Internet of Things, and biomedical fields [8,9,10,11,12,13]. As an essential component of soft electronics, soft electronic sensors are commonly used to detect mechanical signals, such as stress, deformation, and vibration, or to analyze physiological signals, including glucose concentration, pH value, or ion concentration. These sensors have wide-ranging applications in biomedical and health monitoring, prosthetics, and emerging fields such as electronic skin and human–machine interfaces.

In recent years, soft electronic pressure sensors have been intensively studied to achieve high sensitivity and fast response times [14,15,16]. These sensors are categorized as resistive [17,18,19], capacitive [20,21,22], and piezoelectric [23,24,25]. For instance, Zeng et al. reported a flexible graphene–polydimethylsiloxane (PDMS) tactile sensor [26]. Inspired by human finger structure, the sensor’s interdigitated electrode design can detect minute pressure signals, perceive microstructures, and distinguish surface topographies with microscale differences. Zhu et al. developed a highly sensitive pressure sensor using nickel-coated carbon fibers embedded in a PDMS substrate [27]. Huang et al. introduced a resistive pressure sensor array with a pyramidal microstructure, particularly suitable for plantar pressure sensing due to its high sensitivity and durability [28].

Compared to other pressure sensors, capacitive sensors stand out for their low hysteresis, simple design, and low power consumption [20]. These sensors typically have a three-layer structure, with a spacing layer sandwiched between two electrodes. Variations in pressure alter the thickness and effective dielectric constant of the spacing layer, changing the capacitance between the electrodes. Sensitivity can be improved by using specialized materials and porous designs. Examples include a hierarchical porous PDMS sensor with a wide pressure range and negligible hysteresis, ideal for detecting finger taps and breathing [22]; a double-sided porous sensor with high resolution [29]; and a porous PDMS sensor with capacitive/ion dual responses, enabling touchless navigation and zooming on electronic maps [30].

In addition to porous structures, various microstructures have been investigated to enhance the performance of capacitive pressure sensors. Ruth et al. developed a capacitive sensor with a pyramid microstructure, achieving high sensitivity for in vitro pulse monitoring [21]. Cao et al. designed a capacitive sensor with a microstructured layer fabricated using mesh fabric as a template. This simple and cost-effective method allows easy control of the microstructure without the need for expensive equipment [31]. This sensor detected human movements such as finger bending, pressing, fist clenching, and swallowing. Electrode modifications have also been employed to improve sensitivity, such as the ultra-high sensitivity capacitive pressure sensor featuring convex microarray electrodes demonstrated by Xiong et al. [15]. This sensor achieved exceptional sensitivity (30.2 kPa^−1^) and a low detection limit (0.7 Pa) and was applied to monitor human biological signals and robotic arm movements.

The aforementioned studies typically focused on sensors with fixed sensitivity, whereas many practical applications require tunable properties. For instance, in industrial settings, robots often need to sort products of varying stiffness with adjustable sensitivity to prevent damage or slippage or to scan materials of different roughness to identify micro-cracks accurately. In healthcare, high sensitivity is essential for monitoring a patient’s heart rate to capture weak signals at rest, while low sensitivity is preferred during movement to minimize noise. Thus, there is a demand for flexible sensors with adjustable sensitivity. In this paper, we developed a soft capacitive pressure sensor with adjustable sensitivity, which utilizes a PDMS channel as the spacing layer. Tunable sensitivity is achieved by injecting different liquids into the channel.

## 2. Materials and Methods

**Figure 1:**
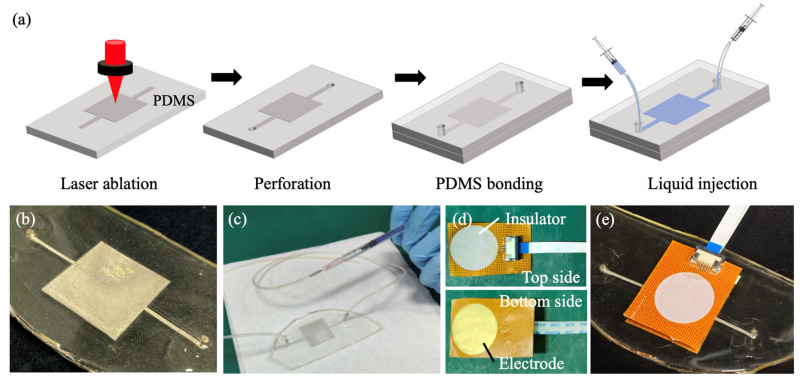
PDMS-based soft capacitive pressure sensor. (**a**) Fabrication process flow, (**b**) PDMS chip with highly rugous PDMS channel, (**c**) liquid injection to tune the pressure sensor, (**d**) electrode with electronic interconnect, and (**e**) integrated pressure sensor.

The spacing layer of the pressure sensor is a PDMS layer with a liquid chamber. First, the base and curing agent of the silicone elastomer (Dow Corning Sylgard 184, Midland, MI, USA) were mixed at a weight ratio of 10:1. Air bubbles trapped in the mixture were removed through degassing, followed by a 3 h heating process at 60 °C to cure the mixture and form a flat PDMS layer. This PDMS layer was then laser ablated, as shown in Figure 1a, resulting in a highly rugous square area with a side length of 25 mm and a straight line with a width of 1 mm crossing the square area. After perforating the two ends of the straight line, the laser-ablated PDMS layer was bonded to another smooth PDMS layer through a plasma treatment process. Only the unablated areas of the two PDMS layers were bonded. In contrast, the square and straight-line areas remained unbonded due to surface roughness, leaving a reservoir area for liquid injection (Figure 1b). The two ends of the straight channel functioned as the inlet and outlet ports for liquid injection, enabling the soft capacitive pressure sensor to achieve adjustable sensitivity by injecting different liquid solutions into the channel (Figure 1c).

Two identical electrode layers were fabricated, consisting of a copper circular pad with a diameter of 25 mm on a PI thin film (Figure 1d). These electrode layers were adhered to the PDMS spacing layer using adhesive tape (Figure 1e). To investigate the sensitivity and tunability of the pressure sensor, various liquid solutions—including a 75% alcohol solution, deionized (DI) water, and a 5% salt water solution—were injected into the rectangular structure within the PDMS layer through the inlet port using a syringe. The outlet port was connected to another empty, fixed syringe, allowing the liquid in the spacing layer to be displaced into the syringe under pressure and flow back when the pressure was released so that no leakage or evaporation was observed. A reference sample was also prepared by injecting air into the rectangular structure.

## 3. Results and Discussion

### 3.1. Sensitivity

**Figure 2:**
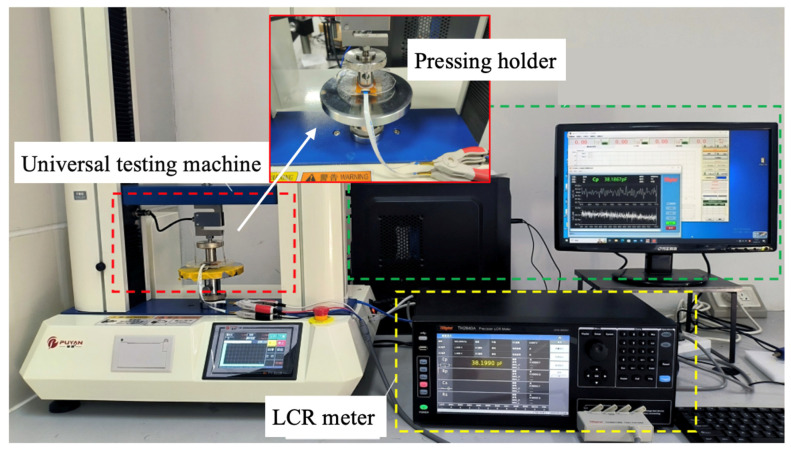
Experimental setup for pressure sensing experiments.

To investigate the sensitivity of the pressure sensor, the pressure was applied to the sensor, and the capacitance between its electrodes was monitored in real time using the experimental setup shown in Figure 2. An electronic universal testing machine (Dongguan Yaofeng, PY-882B, Dongguan, China) was employed to apply a controllable force perpendicular (normal) to the surface of the pressure sensor through a pressing holder. An LCR meter (Changzhou Tonghui, TH2840A, Changzhou, China) was used to measure the sensor’s real-time capacitance. A computer was connected to the electronic universal testing machine and the LCR meter to synchronize force control and capacitance measurement.

**Figure 3:**
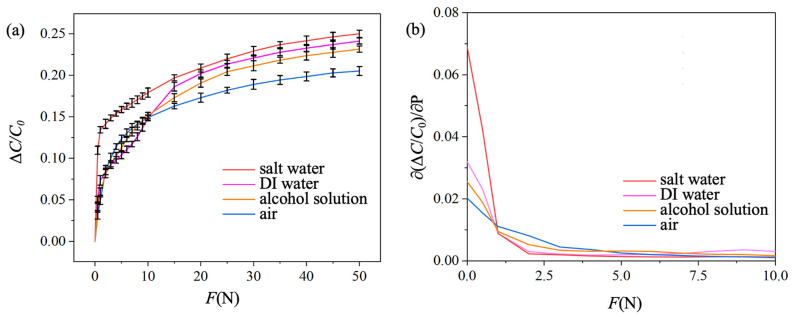
(**a**) Relative capacitance change and (**b**) sensitivity at different exerted forces for pressure sensors injected with different fluid media.

The sensitivity of the pressure sensor was investigated by evaluating the relative capacitance change of the sensor under a normal pressing force, *F*, ranging from 0 to 50 N. The diameter of the pressing holder was 2 cm, which is smaller than the electrode diameter. Therefore, the effective area of the electrode, *A*, was 3.14 × 10^−4^ m^2^, and the corresponding pressure, P=F/A, ranged from 0 to 159 kPa. The initial capacitance is denoted as $C_{0}$, and the measured relative capacitance changes of the sensor under different pressures is $∆C/C_{0}$. Three fabricated pressure sensors were investigated, and the mean values are plotted in Figure 3a, with the error bars representing the standard deviation. The blue, yellow, pink, and red curves represent the relative capacitance changes for the pressure sensor injected with air, alcohol solution, DI water, and salt water, respectively. The measured results exhibit similar behavior across all solutions, which can be analyzed within three distinct force ranges. When the applied force is between 0 and 1 N, the sensor’s capacitance increases sharply as the force rises. The capacitance increases gradually with increasing force for forces between 10 and 50 N. In the 1 to 10 N range, the increasing rate of capacitance falls between the other two ranges.

The sensor sensitivity, $S=\frac{\partial \left(\Delta C/C_{0}\right)}{\partial P}$, was calculated for each force range, along with the coefficient of determination obtained through linear regression analysis to evaluate the linearity of the sensitivity. The calculated sensitivity for each force range is presented in Table 1. S and σ are the mean sensitivity and standard deviation, respectively, based on three experiments. In the 0 to 1 N force range, a mean sensitivity of 1.54 × 10^−2^ kPa^−1^ was achieved when the PDMS channel was injected with air. The mean sensitivity increased to 4.22 × 10^−2^ kPa^−1^ with salt water in the PDMS channel, representing approximately a 2.73-fold improvement. In the 1 to 10 N force range, the mean sensitivity doubled from 1.50 × 10^−3^ kPa^−1^ to 3.04 × 10^−3^ kPa^−1^ when the injected liquid was changed from salt water to ethyl alcohol. These results demonstrate that the sensitivity can be significantly adjusted by injecting different liquids within specific force ranges. For forces ranging from 10 to 50 N, however, there was no significant difference in sensitivity between sensors injected with different liquids. Additionally, the coefficients of determination ($R^{2}$) across all three force ranges exceeded 0.85, indicating good sensing linearity within each range.

To better understand the relationship between sensitivity S and the applied force *F*, $S=\frac{\partial \left(\Delta C/C_{0}\right)}{\partial P}$ at different *F* values was calculated and plotted in Figure 3b. In the small force range from 0 to 1 N, the sensor with air injection exhibited the lowest sensitivity, likely because air has a low relative dielectric constant of 1. The sensitivity increased for the sensor injected with alcohol and further increased for the sensor injected with DI water. This trend could be attributed to the higher dielectric constants of the 75% alcohol solution and DI water, which are 28 and 78, respectively. The highest sensitivity was observed in the sensor injected with salt water, likely due to its electrolyte properties. The sensor injected with air exhibited the highest sensitivity in the force range between 1 and 4 N. This could be because air is easily compressible by higher forces, whereas the sensors injected with the other three liquids were harder to compress.

### 3.2. Limit of Detection

**Figure 4:**
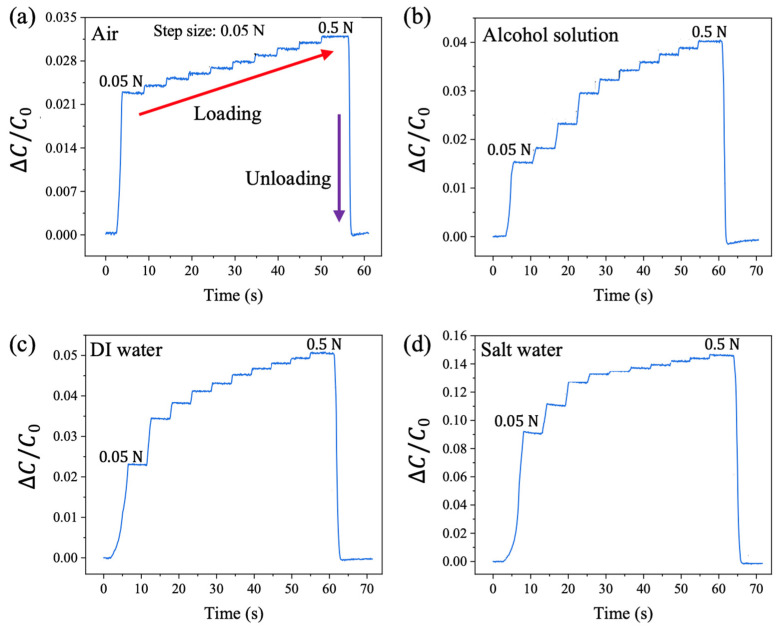
Limit of detection investigation with different fluid media in the PDMS channel. (**a**) Air, (**b**) alcohol solution, (**c**) DI water, and (**d**) salt water.

The limit of detection for this sensor was investigated by using a universal testing machine to apply a force of 0.05 N normally to the sensors at a speed of 0.15 mm/s. The force was gradually increased in increments of 0.05 N, with each increment maintained for 5 s until the force reached 0.5 N. The sensor’s capacitance was measured in real time, and the relative capacitance changes for sensors with different liquid injections were plotted in Figure 4. An increase in the relative capacitance change was observed at each incremental force step, demonstrating a robust detection limit of 0.05 N for the pressure sensor.

Specifically, when no liquid was present in the sensor, the relative capacitance change increased by approximately 0.2% for every 0.05 N increment in the applied pressing force (Figure 4a). When the sensor was injected with an alcohol solution (Figure 4b), the relative capacitance change increased by about 0.5% per 0.05 N increment. In comparison, the relative capacitance change rose by 1.3% and 2% for sensors injected with DI water and salt water, respectively (Figure 4c,d), as the loading force increased from 0.05 N to 0.1 N. These results indicate excellent performance in detecting small forces below 0.1 N.

### 3.3. Response and Relaxation Time

**Figure 5:**
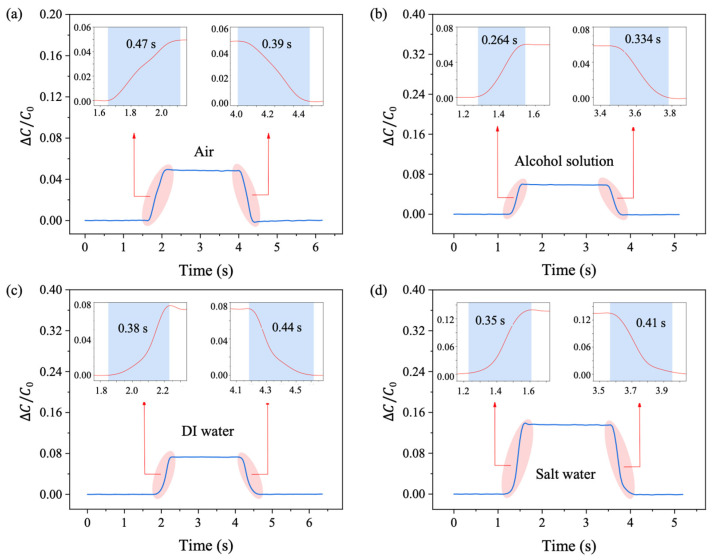
Response time and relaxation time investigation with different fluid media in the PDMS channel. (**a**) Air, (**b**) alcohol solution, (**c**) DI water, and (**d**) salt water.

The response time and relaxation time of the sensor were then investigated. The electronic universal testing machine was programmed to apply a 1 N force to the sensor at an initial speed of 1.5 mm/s, maintaining the force for 2 s before releasing it. The recorded relative capacitance changes are shown in Figure 5. The measured response time of the sensor injected with an alcohol solution was 0.26 s, which is significantly shorter than that of the sensors injected with DI water, salt water, and air, measuring 0.47 s, 0.38 s, and 0.35 s, respectively. Thus, injecting alcohol into the PDMS channel improved the response time by up to 81% compared to the air-injected sensor. During the relaxation phase, the times were 0.39 s, 0.33 s, 0.44 s, and 0.41 s for sensors injected with air, alcohol solution, DI water, and salt water, respectively. Among these, the sensor injected with the alcohol solution also demonstrated superior performance compared to the others.

### 3.4. Hysteresis Analysis

**Figure 6:**
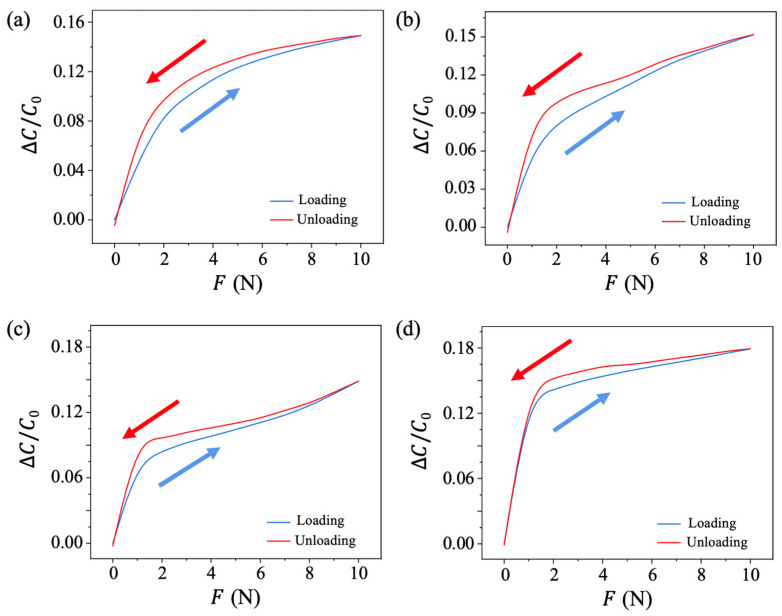
Hysteresis analysis with different fluid media in the PDMS channel. (**a**) Air, (**b**) alcohol solution, (**c**) DI water, and (**d**) salt water.

Hysteresis is another critical parameter of the pressure sensor that evaluates the difference between the sensor’s output readings during pressure loading and unloading. In this study, the universal testing machine was set to apply a force ranging from 0 to 10 N to the sensor injected with different liquid solutions at a pushing speed of 0.33 mm/s. The force was progressively loaded onto the sensor and then unloaded in the opposite direction while the LCR meter monitored real-time capacitance. The relative capacitance changes of pressure sensors injected with different liquid solutions during the hysteresis testing process are shown in Figure 6.

The hysteresis curve follows the blue line during force loading and the red line during force unloading. In all cases, the capacitance change returned to its original value after completing the loading and unloading cycles. However, the blue and red curves did not overlap, indicating significant hysteresis. This phenomenon originates from the fact that during unloading, the sensor remained deformed momentarily due to the residual effect of the previously applied larger force caused by the reaction time delay. This explains why the loading curve is below the unloading curve in the hysteresis graph.

The hysteresis value, defined as the maximum value of $\left(\Delta C_{unload}-\Delta C_{load}\right)/C_{0}$ during the loading and unloading process, was measured as 1.7%, 2.3%, 1.8%, and 1.5% for sensors injected with air, alcohol solution, DI water, and salt water, respectively. Among these, the pressure sensor injected with salt water exhibited the lowest hysteresis value.

### 3.5. Repeatability

**Figure 7:**
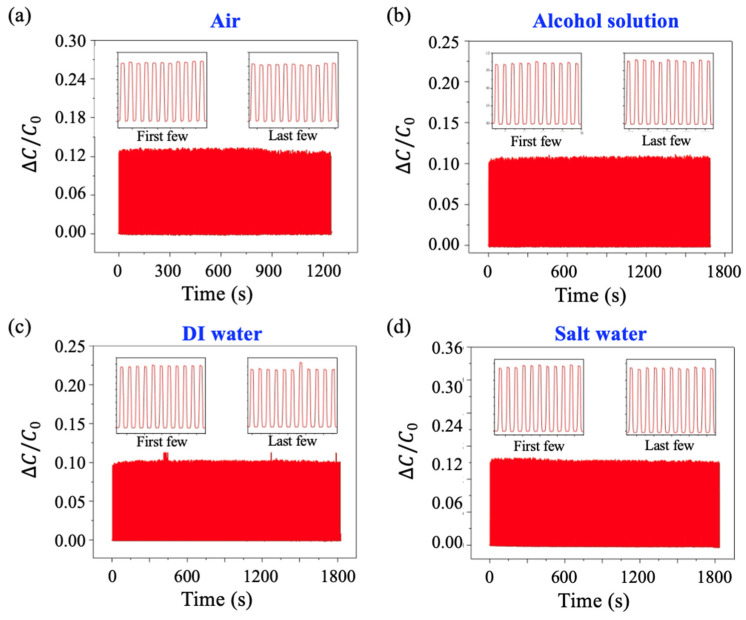
Repeatability investigation with different fluid media in the PDMS channel. (**a**) Air, (**b**) alcohol solution, (**c**) DI water, and (**d**) salt water.

The repeatability of the pressure sensor with different injected liquid solutions was investigated by subjecting it to a 5 N force applied and removed over 500 cycles (Figure 7). The insets display the zoomed-in measurements of the first and last few cycles. The relative changes in capacitance for each sensor during the test remained approximately 0.12, 0.10, 0.08, and 0.16 for sensors injected with air, alcohol solution, DI water, and salt water, respectively. The consistent capacitance changes throughout the testing process indicate that all pressure sensors demonstrated good repeatability. Slight fluctuations were observed, likely caused by mechanical vibrations during the experiment.

### 3.6. Applications

During the capacitance measurement using the universal testing machine, we observed that the sensor’s capacitance changed when the pressing holder approached the sensor, even before making contact. This phenomenon occurs because the electric field is not confined solely to the spacing layer when a voltage is applied between the two electrodes of the pressure sensor. The electric field also extends outside the spacing layer and can be influenced by nearby materials. Consequently, the effective dielectric constant of the capacitor increases, resulting in a rise in sensor capacitance.

**Figure 8:**
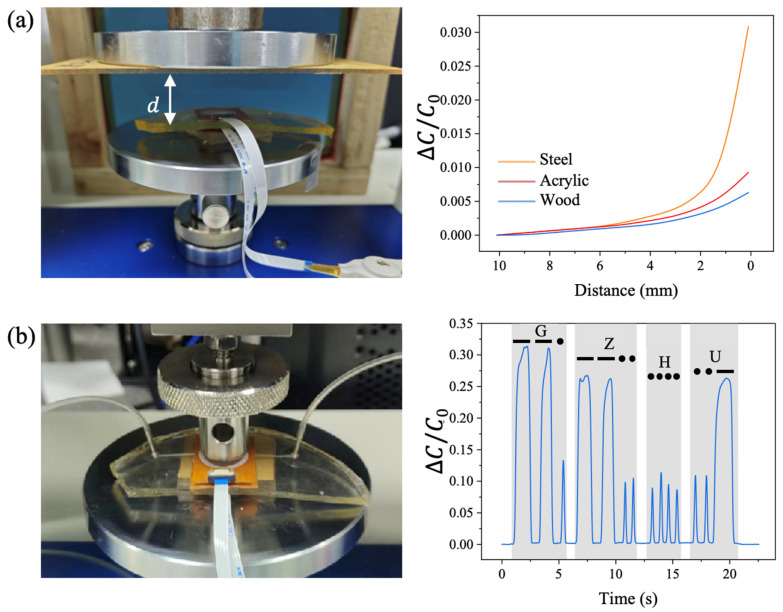
Sensing applications. (**a**) Material property, and (**b**) Morse code representation (‘—’ represents a dash and ‘⋅ ’ represents a dot).

Inspired by this observation, we investigated this property by adhering different materials to the pressing holder. Capacitance changes between the electrodes were measured while varying the distance *d* between the adhered material and the sensor, as shown in Figure 8a. DI water was injected into the sensor, and the adhered square-shaped testing materials included a wood board, acrylic sheet, and iron plate, each with a side length of 20 mm and thickness of 1 mm. Initially, these materials were positioned 10 mm away from the sensor, and the initial capacitance ($C_{0}$) was recorded. As the materials moved closer to the sensor until slight contact was made, the measured capacitance between the electrodes increased with the relative capacitance changes. Capacitance showed a slight increase when the distance between the materials and the sensor was in the range of 10 mm to 6 mm. All materials exhibited similar capacitance changes in this region, likely because the materials were far enough from the sensor that the capacitance was primarily determined by the sensor’s intrinsic properties. When the distance dropped below 6 mm, the capacitance changes began to differ for each material, indicating the materials’ influence on sensor capacitance. At distances below 2 mm, the capacitance increased significantly when the iron plate was adhered to the pressing holder, distinguishing it from the other materials. At distances below 1 mm, the capacitance increase was more pronounced for the acrylic sheet adhered to the pressing holder compared to the wood board. Thus, the three materials could be robustly identified based on their distinct capacitance effects.

The pressure sensor can also be used to represent dash and dot signals in a Morse code system by controlling the pressing duration on the sensor. The experimental setup is shown in Figure 8b. A long-duration press on the sensor represents a “dash” in Morse code, while a short-duration press represents a “dot”. Based on the international Morse code chart, specific sequences of letters can be expressed by pressing the sensor with controlled durations. For instance, two long-duration presses on the sensor injected with DI water generate two pulses of capacitance change lasting for 1 s each, while a short-duration press results in a pulse lasting less than half a second. This signal corresponds to a dash–dash–dot code representing the letter “G” in Morse code. Similarly, the letters “Z”, “H”, and “U” can be expressed, forming the abbreviation for Guangzhou University.

## 4. Conclusions

In conclusion, a soft capacitive pressure sensor with adjustable sensitivity is demonstrated based on the microfluidic PDMS technology. The PDMS channel is easily fabricated using the laser ablation method. We employed liquid solution as the dielectric layer for tunability in terms of sensitivity. The soft capacitive pressure sensor shows a 2.73-fold increment in sensitivity by replacing the fluid medium from air to salt water and a good sensing linearity with a determination coefficient higher than 0.85. The soft capacitive pressure sensor achieved a limit of detection as low as 0.05 N, a response and relaxation time < 0.5 s, a hysteresis value of <2.5%, and good repeatability. We employed the sensitive and fast-responding soft capacitive pressure sensor in measuring materials’ properties and demonstrating the adaptation of the Morse code system. We envisage that the tunable soft capacitive pressure sensor with good linearity, limit of detection, and fast response time has broad applications in wearable devices, material metrology, healthcare point-of-care devices, etc.

In conclusion, a soft capacitive pressure sensor with adjustable sensitivity was developed based on microfluidic PDMS technology. The PDMS channel was fabricated easily using the laser ablation method. Liquid solutions were employed as the dielectric layer to adjust the sensitivity. The soft capacitive pressure sensor exhibited a 2.73-fold increase in sensitivity when the fluid medium was replaced from air to salt water, along with good sensing linearity indicated by a determination coefficient exceeding 0.85. The sensor achieved a detection limit as low as 0.05 N, a response and relaxation time of less than 0.5 s, a hysteresis value of less than 2.5%, and excellent repeatability. The sensitive and fast-responding soft capacitive pressure sensor was utilized to measure material properties and demonstrate its integration into the Morse code system. We foresee that this tunable soft capacitive pressure sensor, with its strong linearity, low detection limit, and rapid response time, has extensive applications in wearable devices, material metrology, healthcare point-of-care devices, and more.

## Figures and Tables

**Table 1:** Sensitivity test calculation data based on different liquid dielectric layers.

Force Range	Sensitivity and Determination Coefficient	Fluids in PDMS Channel			
		Salt Water	DI Water	Ethanol	Air
0 to 1 N	S (kPa^−1^)	4.22 × 10^−2^	2.31 × 10^−2^	1.89 × 10^−2^	1.54 × 10^−2^
	σ	1.8 × 10^−3^	3.6 × 10^−4^	5.4 × 10^−4^	3.6 × 10^−4^
	$R^{2}$	0.882	0.953	0.96	0.969
1 to 10 N	S (kPa^−1^)	1.50 × 10^−3^	2.43 × 10^−3^	3.04 × 10^−3^	3.03 × 10^−3^
	σ	3.6 × 10^−5^	3.6 × 10^−5^	4.8 × 10^−5^	3.6 × 10^−5^
	$R^{2}$	0.986	0.988	0.972	0.866
10 to 50 N	S (kPa^−1^)	5.39 × 10^−4^	6.26 × 10^−4^	5.89 × 10^−4^	4.26 × 10^−4^
	σ	0	9.1 × 10^−5^	0	1.8 × 10^−5^
	$R^{2}$	0.945	0.85	0.906	0.943

## Data Availability

Data are contained within the article.

## Abbreviations

The following abbreviations are used in this manuscript:

PDMS	polydimethylsiloxane
CNT	carbon nanotube
PI	polyimide
DI water	deionized water

## References

[1] Kim J., Kumar R., Bandodkar A.J., Wang J. (2017). Advanced Materials for Printed Wearable Electrochemical Devices: A Review. Adv. Electron. Mater..

[2] Xu H.H., Yin L., Liu C., Sheng X., Zhao N. (2018). Recent Advances in Biointegrated Optoelectronic Devices. Adv. Mater..

[3] Wang Z., Xu X.Y., Tan R.J., Zhang S., Zhang K., Hu J.L. (2024). Hierarchically Structured Hydrogel Composites with Ultra-High Conductivity for Soft Electronics. Adv. Funct. Mater..

[4] Liu S.Q., Li Y.Z., Wen J., Shen Z.X., Meng Q., Liu Q., Yang F., Zheng S.Y., Li J.G., Sun Z.Y. (2024). Versatile Stretchable Conductor with Exceptional Resilience and Rapid Rebound Capabilities: Toward Sustainable and Damage-Resistant Soft Electronics. Adv. Funct. Mater..

[5] Liu Y.X., Liu J., Chen S.C., Lei T., Kim Y., Niu S.M., Wang H.L., Wang X., Foudeh A.M., Tok J.B.H. (2019). Soft and elastic hydrogel-based microelectronics for localized low-voltage neuromodulation. Nat. Biomed. Eng..

[6] Lv J., Thangavel G., Xin Y.Y., Gao D., Poh W.C., Chen S.H., Lee P.S. (2023). Printed sustainable elastomeric conductor for soft electronics. Nat. Commun..

[7] Vo N.T.P., Nam T.U., Jeong M.W., Kim J.S., Jung K.H., Lee Y., Ma G.R., Gu X.D., Tok J.B.H., Lee T.I. (2024). Autonomous self-healing supramolecular polymer transistors for skin electronics. Nat. Commun..

[8] Tordi P., Ridi F., Samori P., Bonini M. (2025). Cation-Alginate Complexes and Their Hydrogels: A Powerful Toolkit for the Development of Next-Generation Sustainable Functional Materials. Adv. Funct. Mater..

[9] Londono C.D., Cones S.F., Deng J., Wu J.J., Yuk H., Guza D.E., Mooney T.A., Zhao X.H. (2024). Bioadhesive interface for marine sensors on diverse soft fragile species. Nat. Commun..

[10] Jiao C.C., Liu J.H., Yan S., Xu Z.W., Hou Z.R., Xu W.L. (2025). Hydrogel-based soft bioelectronic interfaces and their applications. J. Mater. Chem. C.

[11] Hu C., Wang L., Liu S.B., Sheng X., Yin L. (2024). Recent Development of Implantable Chemical Sensors Utilizing Flexible and Biodegradable Materials for Biomedical Applications. ACS Nano.

[12] Wang S.Y., Song X.P., Xu J., Wang J.Z., Yu L.W. (2025). Flexible silicon for high-performance photovoltaics, photodetectors and bio-interfaced electronics. Mater. Horizons.

[13] Zhang Q., Zhao G.Y., Li Z.Y., Guo F., Huang Y., Guo G.H., Wang J.C., Zhou J.K., Chow L., Huang X.C. (2024). Multi-functional adhesive hydrogel as bio-interface for wireless transient pacemaker. Biosens. Bioelectron..

[14] Luo Y.S., Shao J.Y., Chen S.R., Chen X.L., Tian H.M., Li X.M., Wang L., Wang D.R., Lu B.H. (2019). Flexible Capacitive Pressure Sensor Enhanced by Tilted Micropillar Arrays. ACS Appl. Mater. Interfaces.

[15] Xiong Y.X., Shen Y.K., Tian L., Hu Y.G., Zhu P.L., Sun R., Wong C.P. (2020). A flexible, ultra-highly sensitive and stable capacitive pressure sensor with convex microarrays for motion and health monitoring. Nano Energy.

[16] Yang W., Li N.W., Zhao S.Y., Yuan Z.Q., Wang J.N., Du X.Y., Wang B., Cao R., Li X.Y., Xu W.H. (2018). A Breathable and Screen-Printed Pressure Sensor Based on Nanofiber Membranes for Electronic Skins. Adv. Mater. Technol..

[17] Kim K.H., Hong S.K., Jang N.S., Ha S.H., Lee H.W., Kim J.M. (2017). Wearable Resistive Pressure Sensor Based on Highly Flexible Carbon Composite Conductors with Irregular Surface Morphology. ACS Appl. Mater. Interfaces.

[18] Vatani M., Lu Y.F., Engeberg E.D., Choi J.W. (2015). Combined 3D Printing Technologies and Material for Fabrication of Tactile Sensors. Int. J. Precis. Eng. Manuf..

[19] He J., Xiao P., Lu W., Shi J.W., Zhang L., Liang Y., Pan C.F., Kuo S.W., Chen T. (2019). A Universal high accuracy wearable pulse monitoring system via high sensitivity and large linearity graphene pressure sensor. Nano Energy.

[20] Yang J., Luo S., Zhou X., Li J.L., Fu J.T., Yang W.D., Wei D.P. (2019). Flexible, Tunable, and Ultrasensitive Capacitive Pressure Sensor with Microconformal Graphene Electrodes. ACS Appl. Mater. Interfaces.

[21] Ruth S.R.A., Beker L., Tran H., Feig V.R., Matsuhisa N., Bao Z.A. (2020). Rational Design of Capacitive Pressure Sensors Based on Pyramidal Microstructures for Specialized Monitoring of Biosignals. Adv. Funct. Mater..

[22] Hwang J., Kim Y., Yang H., Oh J.H. (2021). Fabrication of hierarchically porous structured PDMS composites and their application as a flexible capacitive pressure sensor. Compos. Pt. B-Eng..

[23] He W., Dai Z.J., Zou K.K., Li X.Y., Hao S.H., Wang H.L. (2023). Flexible piezoelectric PVDF nanofiber film sensor by blow spinning. Sci. China-Technol. Sci..

[24] Chen Y.L., Pu X.X., Xu X.Y., Shi M.H., Li H.J., Wang D. (2023). PET/ZnO@MXene-Based Flexible Fabrics with Dual Piezoelectric Functions of Compression and Tension. Sensors.

[25] Cao C., Zhou P., Wang J.Q., Liu M.X., Wang P., Qi Y.J., Zhang T.J. (2024). Ultrahigh sensitive and rapid-response self-powered flexible pressure sensor based on sandwiched piezoelectric composites. J. Colloid Interface Sci..

[26] Wang H.H., Cen Y.M., Zeng X.Q. (2021). Highly Sensitive Flexible Tactile Sensor Mimicking the Microstructure Perception Behavior of Human Skin. ACS Appl. Mater. Interfaces.

[27] Jiang Y., Liang F., Li H.Y., Li X., Fan Y.J., Cao J.W., Yin Y.M., Wang Y., Wang Z.L., Zhu G. (2022). A Flexible and Ultra-Highly Sensitive Tactile Sensor through a Parallel Circuit by a Magnetic Aligned Conductive Composite. ACS Nano.

[28] Zhang P., Wang X.F., Li Y.X., Zhang K., Huang L.S. (2023). Plantar Pressure Monitoring System Based on a Flexible Pressure Sensor Array for Human Walking Feature Recognition. IEEE Trans. Electron Devices.

[29] Yu Q.Y., Zhang J. (2023). Flexible Capacitive Pressure Sensor Based on a Double-Sided Microstructure Porous Dielectric Layer. Micromachines.

[30] Wang H.L., Chen T.Y., Zhang B.J., Wang G.H., Yang X.D., Wu K.L., Wang Y.F. (2023). A Dual-Responsive Artificial Skin for Tactile and Touchless Interfaces. Small.

[31] Cao S.J., Li R.Q., Panahi-Sarmad M., Chen T.J., Xiao X.L. (2022). A Flexible and Highly Sensitive Capacitive Pressure Sensor with Microstructured Dielectric TPU Layer Based on Mesh Fabric as Template. IEEE Sens. J..

